# Automatic Diagnosis of Left Valvular Heart Disease Based on Artificial Intelligence Stethoscope

**DOI:** 10.1016/j.jacadv.2025.101993

**Published:** 2025-08-20

**Authors:** Ziwei Zhou, Kaipeng Xie, Yiquan Huang, Wenjing Zhang, Bingzhen Li, Junqi Zhong, Xinxue Liao, Changhong Wang, Xiaodong Zhuang

**Affiliations:** a Department of Cardiology, The First Affiliated Hospital of Sun Yat-sen University, Guangzhou, China; b NHC Key Laboratory of Assisted Circulation, Sun Yat-sen University, Guangzhou, China; c School of Biomedical Engineering, Shenzhen Campus of Sun Yat-Sen University, Shenzhen, China

**Keywords:** artificial intelligence, machine learning, stethoscope, valvular heart disease

## Abstract

**Background:**

Valvular heart disease (VHD) remains underdiagnosed and results in serious complications. Early screening for VHD facilitates enhanced clinical management.

**Objectives:**

This study aim to develop an artificial intelligence–based stethoscope model for detecting left-sided VHD, including aortic stenosis, aortic regurgitation, mitral stenosis, and mitral regurgitation.

**Methods:**

Using an electronic stethoscope, we recorded heart sounds from derivation group to construct a machine learning algorithm. Then, the algorithm was tested on a testing group. Echocardiography was referred as the gold standard. Model performance was assessed using area under the receiver-operating characteristic (AU-ROC).

**Results:**

A total of 514 patients were included in the final analyses (304 in the algorithm training group and 210 in the result testing group). The diagnostic performance of machine learning model was as follows: aortic stenosis (AU-ROC: 0.7621), aortic regurgitation (AU-ROC: 0.7075), mitral stenosis (AU-ROC: 0.6426), mitral regurgitation (AU-ROC: 0.7906), and left-sided VHD (AU-ROC: 0.8541; sensitivity 83.07%, specificity 78.26%). When applied to the testing group, the sensitivity, specificity, and AU-ROC for identifying left-sided VHD were 70.00%, 73.68%, and 0.7554, respectively.

**Conclusions:**

Artificial intelligence–based stethoscope is capable of diagnosing left-sided VHD accurately and may make routine screening for VHD more practical.

Valvular heart disease (VHD) is common and its prevalence is rapidly increasing worldwide, with a prevalence of 13.3% in the 75 years and older group.^1^^,^^2^ Left-sided VHD, such as aortic stenosis (AS), aortic regurgitation (AR), mitral stenosis (MS), and mitral regurgitation (MR), are mainly contributors to VHD-related morbidity and mortality.^3^, ^4^, ^5^ VHD has a protracted asymptomatic period.^6^ As a result, a substantial portion of patients with VHD are only diagnosed until the later stages of the disease, with a series of complications.^7^^,^^8^ Thus, it is crucial to early recognize VHD.

The stethoscope has been used for the auscultation of VHD for over 200 years.^9^ However, its diagnostic accuracy is affected by obesity, emphysema, and environmental noise and is highly dependent on clinical professional experience.^10^^,^^11^ Echocardiography is the gold standard for diagnosing VHD, but it requires trained sonographers and experienced cardiologists to acquire and interpret the images, as well as time-consuming and high cost.^12^ Reliance on echocardiography over the past decades has led to underuse of auscultation by the clinician.^13^ It should not be ignored that cardiac auscultation plays an extremely important auxiliary role in the diagnosis of VHD.^14^

Artificial intelligence (AI)–based stethoscope has shown great promise in medicine and may serve as a feasible solution for physicians' inexperience.^12^^,^^14^, ^15^, ^16^ Prior studies have focused on detection of isolated AS or cardiac murmur using processed heart sound signals.^12^^,^^14^ Of note, AR, MS, MR, and even mixed vascular diseases are also highly prevalent in clinical settings. Therefore, we used AI-based stethoscope with the following aims: 1) to accurately identify AS, AR, MS, and MR individually based on machine learning analysis of raw heart sound data collected in clinical settings; and 2) to detect the presence of aortic valve diseases (AS or AR), mitral valve diseases (MS or MR), and left-sided VHD (any of AS, AR, MS, and MR) from a single unified prediction.

## Methods

### Study population

**Figure 1:**
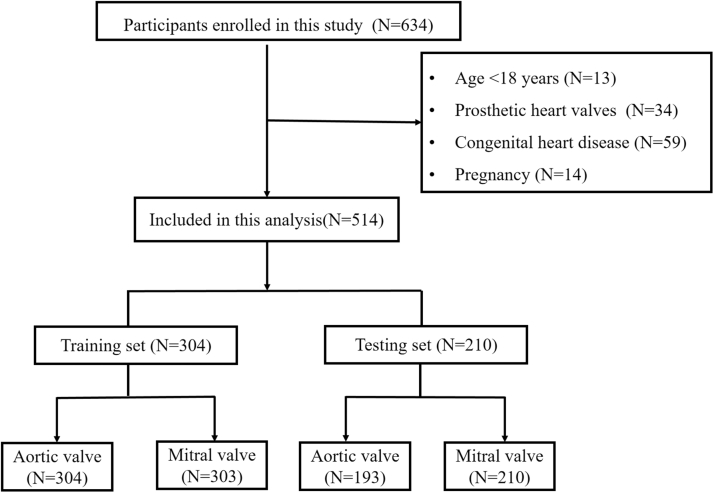
Flowchart for the Selection of the Study Population Flowchart for the selection of the Stethoscope study participants included in this study.

Stethoscope study (NCT06386016) prospectively utilized data from inpatients at the First Affiliated Hospital of Sun Yat-Sen University from May 8, 2023, to March 25, 2024, all of whom had complete echocardiographic data and stethoscope recordings (n = 634). The following patients were excluded from the study (n = 120): 1) age <18 years; 2) prosthetic heart valves or congenital heart disease (other than a bicuspid aortic valve); and 3) pregnancy. Data from the remaining 514 patients were retrospectively analyzed (Figure 1). The study was divided into training (n = 304) and testing (n = 210) phase according to the chronological order of enrollment, setting December 1, 2023 as the time cutoff point (Figure 1). The protocol was conducted in accordance with the Declaration of Helsinki and was also approved by Independent Ethics Committee for Clinical Research and Animal Trials of the First Affiliated Hospital of Sun Yat-Sen University. Informed consent was obtained from all participants.

### Definition of VHD

Experienced cardiologists performed echocardiography on the patients and classified the valve conditions according to the latest clinical guidelines, including normal, stenosis, and regurgitation.^17^^,^^18^ The definitions of AS are as follows: 1) mild AS was defined as peak aortic jet velocity (Vmax) 2.6-2.9 m/s, mean aortic pressure gradient <20 mm Hg, or aortic valve area (AVA) >1.5 cm^2^; and 2) moderate-to-severe AS was defined as peak aortic jet velocity ≥3 m/s, mean aortic pressure gradient ≥20 mm Hg, or AVA ≤1.5 cm^2^. The definitions of AR are as follows: 1) mild AR was defined as effective regurgitant orifice area (EROA) <10 mm^2^, vena contracta width <3 mm, or pressure half-time >500 ms; and 2) moderate-to-severe AR was defined as effective regurgitant orifice area (EROA) ≥10 mm^2^, vena contracta width ≥3 mm, or pressure half-time ≤500 ms. The definitions of MS are as follows: 1) mild MS was defined as mitral valve area (MVA) 1.5 to 2.0 cm^2^, mean mitral pressure gradient <5 mm Hg, pulmonary pressures <30 mm Hg; and 2) moderate-to-severe MS was defined as MVA ≤1.5 cm^2^, mean mitral pressure gradient ≥5 mm Hg, pulmonary pressures ≥30 mm Hg. The definitions of MR are as follows: 1) mild MR was defined as EROA <20 mm^2^, vena contracta width <3 mm; and 2) moderate-to-severe MR was defined as EROA ≥20 mm^2^, vena contracta width ≥3 mm. The collection protocol defined mild or more severe stenosis and moderate or more severe regurgitation detected by echocardiography as valvular disease.

### Heart sound signal collecting and processing

**Figure 2:**
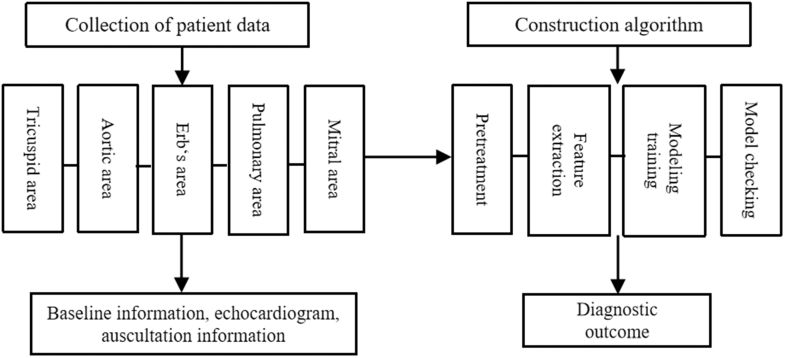
Heart Sound Signals Processing and Algorithm Model Development The development of the algorithm be divided into the following key steps: heart sound signal acquisition (5 auscultation areas), baseline data processing, algorithmic model construction, and diagnostic outcome generation.

Well-trained researchers utilized the smartho-D2 electronic stethoscope to auscultate participants in the quiet clinical environment within 24 hours following the formal release of echocardiographic interpretations, sequentially at the pulmonary valve auscultation area (second intercostal space at the left sternal edge), the aortic valve auscultation area (second intercostal space at the right sternal edge), the Erb's area (third intercostal space at the left sternal edge), the tricuspid valve auscultation area (fourth intercostal space at the left sternal edge), and the mitral valve auscultation area (apical auscultation area) (Figure 2). Heart sounds were recorded for 15 seconds at each auscultation area and output in the CHO file format, which was then converted to WAV format by the Affiliated Tongji Hospital of Huazhong University of Science and Technology.

### Algorithm construction

The construction of heart sound signal processing algorithms is as follows: data preprocessing, feature extraction, model training, and testing. First, preprocessing algorithms are used to enhance the data and filter out noise from the untreated heart sound signals; then, features of the heart sound signals are extracted, and the feature set is input into the machine learning model. Finally, the performance of the model is evaluated based on indicators such as accuracy, specificity, sensitivity, and AUC of the diagnostic results (Figure 2).

Step one: Input the raw data into the preprocessing algorithm; apply normalization, pre-emphasis, and wavelet transform to the untreated heart sound signals for data enhancement and noise filtering. Normalization primarily functions to standardize the samples into a standard range; pre-emphasis is intended to preserve the low-frequency components of the signal while increasing the energy of the high-frequency components, eliminating excessive attenuation of high frequencies by the channel; wavelet transform mainly involves the decomposition and reconstruction of the signal, aiming to reduce audio noise while preserving as much audio information as possible.

**Figure 3:**
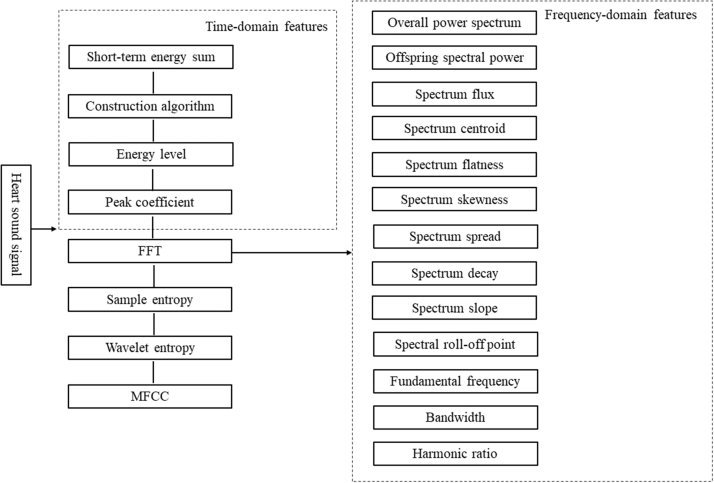
Feature Analysis and Extraction of Audio Signal Characteristics Audio signal characteristics are extracted from the time domain, frequency domain, and other features. FFT = fast Fourier transform; MFCC = Mel-Frequency Cepstral Coefficient.

Step two: After preprocessing, perform feature extraction on the audio. Next, divide the audio into sliding windows with a frame length of 0.4 seconds and a shift of 0.2 seconds. Various features are then extracted in the time domain, frequency domain, cepstrum domain, and nonlinear domain. Time domain features include short-time energy sum, short-time average zero-crossing rate, energy level, and peak coefficient. For spectral features, this paper employs a sliding window approach to perform short-time Fourier transform, segmenting the signal into a series of segments that can be considered stationary (ie, framing), and applies Fourier transform to each frame. The following frequency domain features are obtained: fundamental frequency, spectral flux, harmonic ratio, bandwidth, peak energy frequency, spectral roll-off point, spectral centroid, spectral flatness, spectral skewness, spectral spread, spectral decay, spectral slope, total spectral power, and sub-band spectral power; cepstrum features are represented by Mel Frequency Cepstral Coefficients; nonlinear features include sample entropy and wavelet entropy (Figure 3). To identify the most informative features across different models, we employed the ReliefF algorithm (an instance-based feature weighting evaluation method) to conduct feature informativeness analysis on all constructed predictive models, and selected the top 4 informative features as representatives.

Step 3: Send the extracted feature set to machine learning algorithms and train the recognition model using leave-one-out cross-validation. Seven algorithmic models are established for AS, AR, MS, MR, aortic valve disease (including AS or regurgitation), mitral valve disease (including MS or regurgitation), and left heart valve disease (including AS and regurgitation, MS and regurgitation).^19^^,^^20^ The machine learning algorithms used in these models are support vector machine and K-nearest neighbors. Details regarding our machine learning approach, including the tuning parameters, are shown in the Supplemental Tables 1 and 2. Each model is trained on the training data set and then predicted on the testing data set to output the diagnostic results for the corresponding valve disease. During the training process, the features input into the model are standardized to reduce the impact of feature scale differences, ensuring that each feature's contribution is relatively balanced in the machine learning process. To avoid the issue of sample imbalance, oversampling algorithms are used to increase the number of rare samples to balance the data set, rather than deleting samples from the abundant categories.

### Statistical analysis

Clinical characteristics in this analysis are presented as the mean ± SD for continuous variables or number (%) for categorical variables. And used 1-way analysis of variance (ANOVA) tests or Kruskal-Wallis tests for continuous values and chi-square tests for categorical variables.

The AI algorithm performance was assessed using area under the receiver-operating characteristic (AU-ROC). The sensitivity, specificity, and accuracy were also used to describe the diagnostic efficacy of the AI system. All statistical analyses were performed using IBM SPSS Statistics version 26.0 and *P* value <0.05 was considered as statistically significant.

## Results

In this study, we performed a comprehensive analysis of the baseline demographic characteristics during the training and testing phases (Table 1).

**Table 1:** Baseline Demographic Characteristics in Training and Testing Phases

	Training Phase				Testing Phase				*P* Value^a^
	All (N = 304)	Left-Side VHD Presence (n = 83)	Left-Side VHD Absence (n = 221)	*P* Value	All (N = 210)	Left-Side VHD Presence (n = 26)	Left-Side VHD Absence (n = 184)	*P* Value	
Age, y	63.5 ± 15.4	68.0 ± 12.8	61.8 ± 16.0	0.001	62.3 ± 13.3	64.6 ± 12.2	62.0 ± 13.5	0.377	0.149
Sex				0.897				0.056	0.762
Male	185 (60.9)	51 (61.4)	134 (60.6)		125 (59.5)	11 (42.3)	114 (62.0)		
Female	119 (39.1)	32 (38.6)	87 (39.4)		85 (40.5)	15 (57.7)	70 (38.0)		
BMI, kg/m^2^	23.2 ± 3.7	23.1 ± 3.0	23.2 ± 3.9	0.763	24.6 ± 3.7	24.8 ± 3.5	24.5 ± 3.7	0.727	<0.001
SBP, mm Hg	122.8 ± 20.5	119.0 ± 19.6	124.3 ± 20.7	0.044	125.7 ± 21.5	127.5 ± 24.1	125.4 ± 21.1	0.651	0.131
DBP, mm Hg	71.8 ± 13.8	68.5 ± 15.6	73.0 ± 12.8	0.013	76.0 ± 13.5	75.2 ± 14.1	76.1 ± 13.5	0.757	0.001
Heart rate, beats/min	75.7 ± 15.7	76.3 ± 16.1	75.4 ± 15.6	0.687	79.6 ± 17.9	81.2 ± 20.5	79.4 ± 17.6	0.627	0.009
Hypertension	154 (50.7)	43 (51.8)	111 (50.2)	0.806	129 (61.4)	14 (53.8)	115 (62.5)	0.396	0.016
Diabetes	89 (29.3)	28 (33.7)	61 (27.6)	0.295	63 (30.0)	5 (19.2)	58 (31.5)	0.200	0.860
CVD	150 (49.3)	40 (48.2)	110 (49.8)	0.806	118 (56.2)	7 (26.9)	111 (60.3)	0.001	0.127
AF	62 (20.4)	18 (21.7)	44 (19.9)	0.732	39 (18.6)	12 (46.2)	27 (14.7)	<0.001	0.609
Lung disease	59 (19.4)	30 (36.1)	29 (13.1)	<0.001	57 (27.1)	5 (19.2)	52 (28.3)	0.332	0.039
Cardiac dysfunction	122 (40.1)	55 (66.3)	67 (30.3)	<0.001	64 (30.5)	9 (34.6)	55 (26.9)	0.624	0.025
Bicuspid aortic valve	3 (1.0)	3 (3.3)	0 (0)	0.005	2 (1.0)	2 (7.7)	0 (0)	<0.001	0.969
AS	22 (7.2)	22 (7.2)		<0.001	7 (3.3)	7 (26.9)		<0.001	0.059
AR	27 (8.9)	27 (32.5)		<0.001	9 (4.3)	9 (34.6)		<0.001	0.045
MS	10 (3.3)	10 (12.0)		<0.001	11 (5.2)	11 (42.3)		<0.001	0.273
MR	47 (15.5)	47 (56.6)		<0.001	13 (6.2)	13 (50.0)		<0.001	0.001
EF	62.5 ± 14.5	54.5 ± 16.5	65.5 ± 12.4	<0.001	64.9 ± 11.1	58.5 ± 10.2	65.8 ± 11.0	0.002	0.339

Values are mean ± SD or n (%).

AF = atrial fibrillation; AR = aortic regurgitation; AS = aortic stenosis; BMI = body mass index; CVD = cardiovascular disease; DBP = diastolic blood pressure; EF = ejection fraction; MR = mitral regurgitation; MS = mitral stenosis; SBP = systolic blood pressure; VHD = valvular heart disease.

a Baseline comparison of training phase and testing phase participants.

In the training phase, the prevalence rates of aortic valve diseases (encompassing stenosis and regurgitation) and MR were higher compared to the testing phase, whereas the incidence of MS was relatively lower. Despite generally aligned demographic profiles between cohorts, slight variations emerged in parameters including hypertension prevalence, body mass index, and heart rate. These differences were not clinically significant and were likely related to distribution variations. Additionally, bicuspid valves were rarely observed in both phases.

### Training phase

The sensitivity, specificity, total accuracy, and AU-ROC in the training phase are presented in Table 2. For aortic valve disease, the overall detection performance is at a high level within the categories of regurgitation and stenosis. Notably, AR exhibits the highest specificity, indicating its dominant effectiveness in distinguishing between diseased and healthy individuals. AS shows a slight advantage in sensitivity, reflecting its reliability in detecting true positive cases. In terms of mitral valve disease, the overall detection performance is also satisfactory, although its performance in the stenosis category is weaker compared to that of the aortic valve, particularly in terms of specificity and sensitivity. Our model demonstrates best diagnostic performance for the detection of MR, with an AU-ROC approaching 80%. Overall, the comprehensive evaluation of left-side VHD reveals relatively high accuracy and specificity, indicating that this category possesses good diagnostic capability in overall detection.

**Table 2:** Results of Training Phases

Heart Valve Disease	Category	Accuracy	Specificity	Sensitivity	AUC
Aortic valve	Disease	0.6875	0.6803	0.7428	0.7386
	Stenosis	0.7467	0.7491	0.7143	0.7621
	Regurgitation	0.7763	0.7895	0.5789	0.7075
Mitral valve	Disease	0.7096	0.6972	0.7692	0.7933
	Stenosis	0.6172	0.6143	0.7000	0.6426
	Regurgitation	0.7591	0.7656	0.7234	0.7906
Left heart valve disease		0.7952	0.7826	0.8307	0.8541

AUC = area under the receiver-operating characteristic curve.

### Testing phase

Table 3 shows the sensitivity, specificity, total accuracy, and AU-ROC in the testing phase. The model demonstrates good overall diagnostic performance for aortic valve disease, with similar sensitivity observed for both AS and AR, indicating a certain level of detection capability. In the assessment of mitral valve disease, there is a slight decrease in overall accuracy and specificity. Furthermore, similar to the training phase, the diagnostic performance for MR remains relatively strong, exhibiting higher sensitivity and a good AU-ROC, suggesting its potential value in clinical detection. Overall, the performance of left-side VHD is superior to that of either aortic or mitral valve disease alone, demonstrating higher accuracy and specificity.

**Table 3:** Results of Testing Phases

Heart Valve Disease	Category	Accuracy	Specificity	Sensitivity	AUC
Aortic valve	Disease	0.6839	0.6850	0.6667	0.7362
	Stenosis	0.7150	0.7165	0.6667	0.6325
	Regurgitation	0.7617	0.7676	0.6250	0.7650
Mitral valve	Disease	0.6571	0.6580	0.6471	0.7399
	Stenosis	0.6714	0.6700	0.7000	0.6483
	Regurgitation	0.7714	0.7778	0.6667	0.7077
Left heart valve disease		0.7325	0.7368	0.7000	0.7554

Abbreviation as in Table 2.

## Discussion

Our results suggest that AI-based stethoscope would be a useful tool in detecting left-sided VHD. In the present analysis, the algorithm could accurately identify patients with left-sided VHD of AS (AU-ROC:0.76); AR (AU-ROC: 0.71); MS (AU-ROC: 0.64); MR (AU-ROC: 0.79); and a composite of any of AS, AR, MS, or MR (AU-ROC: 0.85) (Central Illustration).

**Central Illustration:**
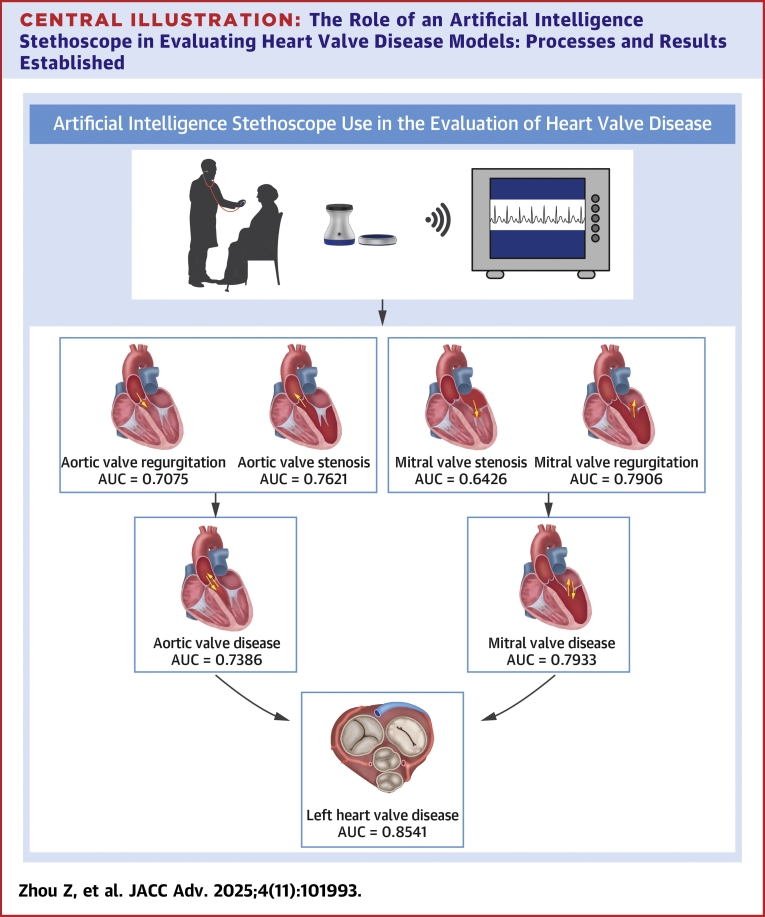
The Role of an Artificial Intelligence Stethoscope in Evaluating Heart Valve Disease Models: Processes and Results Established AUC = area under the receiver-operating characteristic curve.

Heart sound analysis has recently been used in detecting VHDs.^12^^,^^14^^,^^15^^,^^21^^,^^22^ Ghanayim et al^14^ found that an AI-based stethoscope with infrasound capabilities can accurately diagnose AS. Chorba et al^12^ assessed the performance of a deep learning algorithm to detect murmurs caused by AS or MR using recordings from a commercial digital stethoscope platform. However, previous studies mostly focused on Western populations. Given that body size difference exits between Chinese and Western populations, there may be a large deviation when these models are applied to Chinese patients. Consistent with a prior study in China,^15^ we also confirmed that analysis of heart sound data using AI-based stethoscope is capable of diagnosing VHD accurately in Chinese patients. Besides that, results in the present analyses extended prior studies by showing that the best diagnostic performance was observed in detecting a composite of any of AS, AR, MS, and MR.

Any of AS, AR, MS, and MR is becoming more prevalent with increasing life expectancy and plays as an important driver of cardiovascular morbidity and mortality.^23^, ^24^, ^25^ Of note, the presence of multivalvular disease is common and may further accelerate cardiac dysfunction.^26^ Thus, it is necessary to treat the 4 left-side valvular lesions as a whole and develop a model to early detect left-side VHD. Transcatheter valve therapies have expanded therapeutic options for patients with left-side VHD, including for those who previously had no viable surgical options.^23^ Despite the high prevalence and improving treatment options for VHD, there is currently no satisfying screening program to improve VHD diagnosis. The traditional screening method for VHD, that is, physical examination, demonstrates poor accuracy and low consistency for aortic or mitral valve disease.^27^ The gold standard for VHD detection, that is, echocardiography, is difficult to widespread implement due to expense and expertise. The process of our AI-based stethoscope model—from the initial waveform recording to determining the presence and severity of valvular disease—takes approximately 91 seconds without manual input or intervention (Supplemental Table 3). Thus, our models may make routine screening for VHD more practical and promote the early identification and diagnosis of patients with left-side VHD.

The exploration of the most informative features across different cardiac valve disease models indicates that these variations may be related to the pathophysiological mechanisms underlying the diseases (Table 4). Firstly, the sub-band spectral power features capture the energy distribution of heart sounds across different frequency bands, with various valve abnormalities potentially leading to abnormal increases or decreases in energy within specific frequency ranges. Mel Frequency Cepstral Coefficients simulate how the human ear perceives sound,^28^ reflecting the method clinicians use to identify pathological murmurs through auscultation. Secondly, due to the more pronounced energy variations caused by aortic valve diseases, such as the harsh systolic murmur characterized by concentrated energy and high intensity resulting from AS,^29^ short-time energy features may be more prominent in the aortic valve disease model. Additionally, mitral valve abnormalities can result in significant changes in the shape of the heart sound spectrum. For instance, MS produces a low-frequency rumbling sound, while MR generates a high-frequency blowing murmur.^30^ This leads to spectral shape features (such as spectral slope and spectral spread) possessing higher informational content. Finally, the greater impact of aortic valve abnormalities on hemodynamics results in more pronounced acoustic characteristics.^18^ Thus, the analysis of the left-sided VHD model suggests that high-information features from the aortic valve region (such as maximum short-time energy) can provide more information than those from the mitral valve region.

**Table 4:** The Four Most Informative Features Among Various Heart Valve Disease Models

Disease Model	Variables (Features)			
Aortic stenosis	MSTE	MFCC-7	SSP-B2	SpSp
Aortic regurgitation	SSP-B1	MFCC-7	MSTE	MFCC-6
Aortic valve disease	MFCC-7	MSTE	SSP-B2	MFCC-4
Mitral stenosis	MFCC-3	SSP-B2	MFCC-11	ShEn
Mitral regurgitation	SSP-B3	MFCC-5	MFCC-11	SpecSl
Mitral valve disease	SSP-B3	SSP-B2	MFCC-5	SpecSl
Left-sided VHD	MFCC-4	MFCC-6	PEF	MSTE

MFCC-n = Mel-Frequency Cepstral Coefficient (n); MSTE = maximum short-time energy; PEF = peak energy frequency; ShEn = Shannon entropy; SpecSl = spectral slope; SpSp = spectral spread; SSP-Bn = sub-band spectral power (band n); VHD = valvular heart disease.

### Study limitations

This study has several limitations. First, only inpatients admitted to cardiac specialty wards were included in our present analysis, so the generalizability of the model need to be validated in the general population. Second, the performance of AI-based stethoscope may be affected by obesity, emphysema, and severity of VHD. Third, the performance of our model was poor in patients with MS due to small sample. However, the smart stethoscope is intended for screening a composite of any of left-side valvular lesions, so misclassification of other cardiac pathologies will not be an issue, and will be resolved using echocardiography.

## Conclusions

AI-based stethoscope can accurately diagnose left-side VHD and may serve as a promising new tool to promote widespread screening for VHD.

## Funding support and author disclosures

This study was supported by the National Natural Science Foundation of China (82370358 to Dr Liao, 62303496 to Dr Wang), Guangdong Basic and Applied Basic Research Foundation (2024A1515013234 to Dr Liao; 2024A1515012356 to Dr Zhuang). All other authors have reported that they have no relationships relevant to the contents of this paper to disclose.
