# Supplementary material for: Automatic Diagnosis of Left Valvular Heart Disease Based on Artificial Intelligence Stethoscope
Source: JACC Adv. 2025 Aug 20;4(11):101993. doi: 10.1016/j.jacadv.2025.101993 (PMC12717554; doi:10.1016/j.jacadv.2025.101993)
Supplement: Supplementary — data [file mmc1.docx]

**Supplemental Table 1.** Detailed description of the machine learning approach using SVM.

| Disease type/SVM parameters | Kernel function | Feature Standardization | Regularization Parameter | Kernel Scale Parameter |
| --- | --- | --- | --- | --- |
| Aortic regurgitation | Linear Kernel | True | 1 | 1 |

SVM: Support vector machine.

**Supplemental Table 2.** Detailed description of the machine learning approach using KNN.

| Disease type/KNN parameters | Neighborhood Size (K-value) | Feature Standardization | Distance Metric | Weight Function |
| --- | --- | --- | --- | --- |
| Aortic stenosis | 15 | True | Euclidean Distance | Equal Weight |
| Aortic valve disease | 80 | True | Euclidean Distance | Equal Weight |
| Mitral stenosis | 140 | True | Euclidean Distance | Equal Weight |
| Mitral regurgitation | 15 | True | Euclidean Distance | Equal Weight |
| Mitral valve disease | 60 | True | Euclidean Distance | Equal Weight |
| left-sided VHD | 25 | True | Euclidean Distance | Equal Weight |

KNN: K-nearest neighbors; VHD,valvular heart disease.

**Supplemental Table 3.** Time required for AI-based stethoscope model to diagnose VHD.

| Steps | Time of diagnosis |
| --- | --- |
| Heart sound signal acquisition | 90 seconds |
| Heart sound signal preprocessing | 0.2 seconds |
| Feature extraction | 0.8 seconds |
| AI model loading and testing | 0.2 seconds |
| Total duration | 91 seconds. |

AI: Artificial intelligence; VHD,valvular heart disease.
